# Verapamil Restores β-Cell Mass and Function in Diabetogenic Stress Models via Proliferation and Mitochondrial Respiration

**DOI:** 10.3390/cells14211695

**Published:** 2025-10-29

**Authors:** Hossein Arefanian, Fatema Al-Rashed, Fawaz Alzaid, Fatemah Bahman, Nermeen Abukhalaf, Halemah Alsaeed, Shihab Kochumon, Michayla R. Williams, Sarah M. Kidwai, Ghadeer Alhamar, Rasheed Ahmad, Fahd Al-Mulla, Ashraf Al Madhoun

**Affiliations:** 1 Department of Immunology & Microbiology, Dasman Diabetes Institute, Dasman 15462, Kuwait; hossein.arefanian@dasmaninstitute.org (H.A.); fatema.alrashed@dasmaninstitute.org (F.A.-R.); fatemah.bahman@dasmaninstitute.org (F.B.); halemah.alsaeed@dasmaninstitute.org (H.A.); shihab.kochumon@dasmaninstitute.org (S.K.); ghadeer.alhamar@dasmaninstitute.org (G.A.); rasheed.ahmad@dasmaninstitute.org (R.A.); 2 Department of Bioenergetics & Neurometabolism, Dasman Diabetes Institute, Dasman 15462, Kuwait; fawaz.alzaid@dasmaninstitute.org (F.A.); drmichayla@gmail.com (M.R.W.); 3 Institut Necker Enfants Malades (INEM), French Institute of Health and Medical Research (INSERM), Immunity & Metabolism of Diabetes (IMMEDIAB), Université de Paris Cité, 75014 Paris, France; 4 Animal and Imaging Core Facility, Dasman Diabetes Institute, Dasman 15462, Kuwait; nermeen.abukhalaf@dasmaninstitute.org (N.A.); sarahmkidwai@hotmail.com (S.M.K.); 5 Department of Translational Research, Dasman Diabetes Institute, Dasman 15462, Kuwait

**Keywords:** verapamil, diabetes, T1D, T2D

## Abstract

Diabetes remains a global health challenge, characterized by persistent hyperglycemia and gradual depletion or impairment of pancreatic β-cells. Current treatments focus on managing glycemic control, but do not mitigate β-cell mass. Verapamil, an FDA-approved calcium channel blocker for hypertension, has shown potential therapeutic action towards β-cells in the context of diabetes. In this study, we investigated the cytoprotective and metabolic efficacy of verapamil on mouse-derived MIN6 β-cells under metabolic and diabetogenic stressors like high glucose, toxins, and an inflammatory cytokine cocktail, as well as investigated a zebrafish model. At safe, non-toxic doses, verapamil elevated the levels of cholecystokinin (CCK), an incretin associated with β-cell preservation and enhanced mitochondrial respiration. Notably, pretreatment and co-treatment of verapamil in the presence of stressors offered substantial protection and preserved mitochondrial function, whereas post-treatment effects were moderate and model dependent. In the zebrafish model, verapamil promoted β-cell recovery and regeneration before, during, and after targeted ablation. The drug seemed to work in several ways: inducing proliferation, reducing stress on β cells, boosting their energy production, and activating survival signals. Together, our data aligned with earlier human clinical trials showing that verapamil administration preserved β-cell mass and function in patients with recent-onset type 1 diabetes. The high efficacy, affordability, and broad mechanisms of action make verapamil a desirable therapeutic candidate for diabetes. Nevertheless, further mechanistic studies and long-term clinical trials are warranted to establish its utility in diabetes management.

## 1. Introduction

Diabetes is a chronic metabolic disorder with significant global health implications, categorized primarily into type 1 diabetes (T1D) and type 2 diabetes (T2D) (American Diabetes Association Professional Practice Committee, 2022) [1,2]. T2D is caused by insulin resistance and progressive β-cell dysfunction, and T1D arises from autoimmune destruction of pancreatic β-cells by cellular and auto-immune attack, leading to insulin deficiency [3,4,5]. Despite considerable progress toward β-cell replacement and preservation, several fundamental complications preclude translation. Immune-mediated injury and chronic rejection remain major barriers after islet transplantation and cell replacement approaches, resulting in appreciable graft function loss over time [6]. In addition, intrinsic β-cell stress responses, notably endoplasmic reticulum (ER) stress, oxidative stress, and maladaptive unfolded protein responses, have been implicated in β-cell dysfunction and dedifferentiation, foiling attempts to restore or maintain functional β-cell mass [7]. These pathophysiological processes can be exacerbated by metabolic insults (glucotoxicity, lipotoxicity) and by pro-inflammatory microenvironments that limit long-term engraftment and insulin secretory function [8]. Clinical trials, finally, exhibit heterogeneity of response to β-cell preservation strategies and demand larger, longer, and more diverse trials to define durable benefit across populations [9,10]. Collectively, these complications underscore the value of investigating interventions, such as small-molecule repurposing with verapamil, which not only protect β-cells acutely but also target ER stress, inflammation, and microenvironmental determinants of β-cell survival and function over the long term.

Current therapies, such as insulin and incretin-based medications, have disadvantages like hypoglycemia, high costs, invasiveness and are generally inadequate in managing the intrinsic β-cell loss [11,12]. Repurposing existing drugs, like the calcium channel blocker (CCB) verapamil, offers a promising alternative for preserving β-cell function and improving glucose homeostasis [13,14].

Verapamil, initially licensed for use in hypertension and angina, also demonstrated unanticipated benefits in controlling diabetes in humans (Verapamil Infarction Trial II-DAVIT II, 1990) [15,16,17]. Subsequent research revealed that verapamil pretreatment of β-cells in rodents prevents toxin-mediated damage, preserves insulin secretion, and reduces hyperglycemia [18,19]. The results formed the basis for exploring the therapeutic utility of verapamil in diabetes.

Recent proteomic and transcriptomic profiling revealed verapamil’s ability to modify β-cell proliferation, redox homeostasis, and anti-apoptotic signaling [14]. We recently showed that verapamil elevates cholecystokinin (CCK), a peptide hormone produced mainly by entero-endocrine cells in the small intestine and, to a lesser extent, by neurons in the brain. CCK is known to regulate digestion and appetite and promote the feeling of fullness. In addition, CCK has also been reported to enhance β-cell mass and prevent the programmed cell death process known as apoptosis [20]. It reduces voltage-dependent anion channels (VDAC1/2), key components of mitochondrial function [20,21]. Furthermore, verapamil also activates the calcium/calmodulin-dependent protein kinase type IV (CaMK4) and Wnt4 signaling, which mediate β-cell proliferation and glucose sensing [22,23].

Notably, verapamil’s effectiveness in maintaining β-cell function has been confirmed by clinical trials [24]. Verapamil treatment decreased insulin needs, delayed β-cell loss, and maintained C-peptide levels in adults with recent-onset T1D [13,14]. Verapamil preserved C-peptide secretion in pediatric T1D patients with no serious side effects [9,25]. Verapamil decreased fasting plasma glucose and improved glucose tolerance in people with type 2 diabetes [10]. Research shows that it also improves insulin sensitivity and lowers hepatic glucose output [26,27]. Verapamil’s ability to reduce the risk of developing diabetes at an early age was further supported by large cohort studies like the INVEST trial [17,28]. Despite promising results, challenges remain, like patient response variability and the requirement for long-term efficacy data [29]. Verapamil’s advantages in T1D may be increased by combining it with immunomodulatory drugs, but its function in pre-diabetes and metabolic syndrome needs more research [30,31,32].

Verapamil represents a paradigm shift in diabetes therapy, transitioning from a cardiovascular drug to a β-cell protector, showing promise in addressing the underlying cause of β-cell loss in diabetes. These mechanisms can be further clarified in preclinical models like zebrafish and a commonly used murine pancreatic beta-cell line (MIN6 cells), opening the door for targeted treatments. Verapamil may become a mainstay in the treatment of diabetes as clinical trials confirm its safety and effectiveness, providing patients all over the world with an affordable and easily accessible option.

In this study, we investigated the protective and regenerative effects of verapamil on pancreatic β-cells under metabolic and diabetogenic stress. Using MIN6 β-cells, we found that verapamil not only enhanced cell viability and proliferation but also improved mitochondrial function and oxidative phosphorylation in the stressed cells. We also identified upregulation of CCK as a potential mediator of verapamil’s protective effects. Additionally, in the zebrafish model, verapamil promoted β-cell recovery following MTZ-induced damage. These findings collectively support verapamil’s role as a β-cell protective and regenerative agent.

## 2. Materials and Methods

### 2.1. Cell Culture and Treatments and Viability Assays

MIN6 cells were kindly provided by Dr. Jun-ichi Miyazaki, School of Medicine Kumamoto University, Japan [33]. The cells were cultured at 37 °C in a humidified incubator with 5% CO_2_ using DMEM containing 15% fetal bovine serum (FBS), 10 mM HEPES, 1 mM sodium pyruvate, 25.2 mM glucose, 2 mM l-glutamine, 50 μg/mL penicillin, 100 μg/mL streptomycin, and 70 μM β-mercaptoethanol (Invitrogen, Waltham, MA, USA), as previously described [33]. Experiments were performed using cells at passages below 25 to confirm consistency. For treatment, fresh preparations of verapamil hydrochloride (152-11-4, Calbiochem, Burlington, MA, USA) dissolved in phosphate-buffered saline (PBS), aliquoted, and used at the concentrations mentioned in the manuscript.

### 2.2. Cell Survival Assessment and Inhibitory Concentration 50% Determination

For viability assays, the MIN6 cells were seeded in a 96-well plate (Costar, High Wycombe, UK) and allowed to adhere for 24 h. The cells were then washed with PBS and exposed to various concentrations of verapamil (1–1000 μM) for 24 or 48 h. Cell viability was assessed using an MTT assay (Trevigen, Minneapolis, MN, USA), as described in the manufacturer’s protocol [20]. Absorbance was recorded using Synergy H4 (BioTek, Winooski, VT, USA), and the data were analyzed with Gen5 software (v2.03, BioTek, Winooski, VT, USA). To determine IC50, viability percentages were calculated relative to the untreated controls. The verapamil dose response curve was generated by GraphPad Prism ver. 9.0 software (Boston, MA, USA), as previously described [20]. Based on these results, concentrations ranging from 1 to 50 μM were selected for subsequent experiments. This concentration range was chosen because it provided clear dose-dependent biological effects while maintaining cell viability above 95%, consistent with our current and previous studies, as well as published literature [20]. The rationale for these doses was further supported by our toxicity assessment, which demonstrated that 50 μM represented the highest concentration, producing significant biological responses with only minimal, non-statistically significant toxicity.

For experiments involving combinatorial stress conditions, the MIN6 cells were exposed to verapamil alone or in combination with diabetologist insults, including 3 mM streptozotocin (STZ, Sigma-Aldrich, Saint Louis, MO, USA) or a pro-inflammatory T1D cytokine cocktail (T1D-cytomix: 50 ng/mL TNF-α, 50 ng/mL IL-1β, and 100 ng/mL INF-γ (R&D Systems, Minneapolis, MN, USA) in high-glucose culture media [20,34].

### 2.3. Immunofluorescence Detection of Ki67

Cell proliferation was assessed using Ki67 immunofluorescence assay [20]. The MIN6 cells were cultured on coverslips in high-glucose DMEM media and 2% free fatty acid bovine serum albumin (BSA, 820024, Sigma, St. Louis, MO, USA). Cells treated with or without verapamil were fixed and permeabilized with 4% paraformaldehyde and 0.5% saponin for 15 min at RT. After blocking in PBS-Tween, the cells were incubated with Ki67 antibodies (1:1000, 15580, Abcam, Waltham, MA, USA) for 2 h at RT. The cells were then washed with PBS-Tween and incubated with secondary Alexa Fluor 488 antibody (1:1000, ab150077, Abcam) for 1 h at RT. Actin filaments were labeled with Phalloidin-iFluor 594 (ab176757, Abcam), and nuclei were counterstained with DAPI–Aqueous, Fluoro-shield (ab104139, Abcam). Images were obtained using a confocal microscope (LSM710, Carl Zeiss, Gottingen, Germany), and proliferation was quantitated by analyzing correlated total cell fluorescence (CTCF) from 10 fields/sample via ImageJ (v2.9.0), as previously described [20].

### 2.4. Western Blotting

Total protein was extracted in RIPA buffer, and Western blotting was performed as previously described [20,35]. After quantification with Bradford assay (Bio-Rad Laboratories, Hercules, CA, USA), 30 μg proteins were denatured and fractionated on 12% SDS-PAGE gel electrophoresis. The proteins were transferred to nitrocellulose membranes (Bio-Rad Laboratories) by electroblotting. The membranes were then blocked for 1 h and propped overnight with primary antibodies: Histone H3 (D1H2; 4499) and β-Actin (8H10D10; 3700 Cell Signaling Technology, Danvers, MA, USA). CCK (PA5-103116, Thermoscientific, Waltham, MA, USA) or PCNA (ab29, Abcam). Following a TBDT wash, the membranes were incubated for 2 h with fluorescent IRDye-680RD (ab216777, Abcam) and IRDye-800RD (ab216772, Abcam), or horseradish peroxidase-linked conjugated secondary antibodies. Supplementary Figure S1 shows the original non-cropped Western blots. Immunofluorescence and chemiluminescence bands were imaged by Molecular Imager ChemiDoc MP Imaging Systems (v6.1) from Bio-Rad Laboratories.

### 2.5. Cell Cycle Analysis

Cell cycle was assessed by flow cytometry and DNA staining by propidium iodide (PI), as previously described [36]. After the experimental intervention, the cells were immobilized, fixed in ice-cold 70% ethanol, and centrifuged at 800× *g*. Then, they were treated with 100 µg/mL RNase and incubated with 40 µg/mL PI for 30 min. DNA content and cell cycle stages were assessed on a flow cytometer (FACSCant, BD Bioscience, San Jose, CA, USA). The cells were gated and analyzed as previously described [20]. PI detection was carried out at 490 nm excitation and 630 nm emission using BD FACSDiva Software 8 (BD Biosciences).

### 2.6. Cell Viability Assays

Verapamil was used at 1, 5, 10, and 50 μM to determine the half-maximal inhibitory concentration (IC50). The cell protection assays were performed at different stages of verapamil administration with and without diabetogenic stressors, STZ, or the T1D-cytomixes as previously described [20]. In this study, the MIN6 cells were stressed by one of two stressors as follows: 1. T1D cytokine cocktail or 2. Streptozotocin (STZ; Sigma-Aldrich, USA). For each experiment, we used a freshly prepared stock solution of STZ dissolved in cold citrate buffer [34]. The pre-, post-, and co-treatment conditions were set as follows: Pretreatment conditions: The cells were pretreated with 1–50 μM of verapamil for 24 h. Then, the cells were washed with 3× PBS and treated with either stressor, as indicated, for another 24 h. Post-treatment condition: The cells were first treated with the stressors for 24 h, and then with 1–50 μM verapamil for another 24 h. Co-treatment condition: The cells were co-treated with verapamil and either stressor for 24 h. Treatments included the MTT assay for cell survival studies.

### 2.7. Seahorse Assay (Metabolic Flux Analysis)

The mitochondrial function was evaluated by measuring the oxygen consumption rate (OCR) using XFe96 Seahorse (Agilent Technologies, Santa Clara, CA, USA), as described by manufacturers [20]. The MIN6 cells were seeded in a Seahorse XF96 microplate (101085-004, Agilent Technologies, Santa Clara, CA, USA). After 24 h, the cells were exposed to diabetogenic insults with or without 50 μM verapamil as pre-, post-, or co-treatments, and untreated cells served as control. Then, OCR assays were assessed using assay media +25.2 mM glucose, and mitochondrial stress was assessed following the protocol described in the Agilent Technologies Seahorse XF Cell Mito Stress Test Kit brochure (103015-100) [20].

### 2.8. Transgenic Zebrafish Larvae Model Experiments

Zebrafish (*Danio rerio*) were bred in the aquarium at the Dasman Diabetes Institute’s Animal Facility under standard conditions: 12 h light/dark cycle and 28 °C RT. All experimental procedures followed the ARRIVE guidelines and standard zebrafish laboratory protocols, and were approved by the Animal Care Ethical Committee (Project #RA-2019-005, 28 January 2020), in agreement with the National Institutes of Health (NIH) Guide for the Care and Use of Laboratory Animals (NIH Publication No. 8023, revised 1978) [20]. We used the Ins:NfsB-mCherry transgenic zebrafish line, kindly provided by Dr. Michael J. Parsons (University of California, Irvine, USA). In this model, the bacterial nitroreductase (NTR) gene, responsible for converting prodrugs like metronidazole (MTZ) into cytotoxic compounds, is fused to the mCherry fluorescent protein, driven by the insulin promoter [37]. MTZ (M3761, Sigma) was prepared as previously described [38]. Larvae were raised in E3-media at 28 °C in a dark incubator. To assess the effects of verapamil on β-cell regeneration, the larvae were monitored from 3 to 6 days post-fertilization (dpf) by measuring mCherry fluorescence intensity. The larvae were divided into five groups: Group 1: untreated larvae (control); Group 2: larvae treated with MTZ (10 mM) at 3 dpf for 24 h; Group 3: larvae pretreated with verapamil (10 µM) for 24 h, then exposed to MTZ (10 mM) for 48 h; Group 4: larvae treated with MTZ (10 mM) at 3 dpf for 24 h, followed by a 48 h recovery period in verapamil (10 µM); and Group 5: larvae co-treated with both verapamil (10 µM) and MTZ (10 mM) for 72 h. The rationale for selecting 10 µM verapamil was based on dose–response testing in zebrafish embryos using a range of concentrations. Among these, 10 µM produced the most pronounced biological effects while exhibiting only minimal, non-statistically significant toxicity.

Whole-body images were acquired using a Discovery V12 Stereo microscope (Zeiss) equipped with an Alexa Fluor 594 red fluorescence filter and Zeiss Zen Blue software (v3.10), as previously described [20]. Images were processed in ImageJ. Processing was automated with a macro written in JavaScript (v.ij154-win-java8). The RGB images were separated into constituent colors to identify and subtract nonspecific fluorescent green channel data from the red channel. An intensity threshold was applied uniformly to all images to select the positive region. The region was measured for average intensity and area to determine mCherry expression (a schematic description is presented in Supplementary Figure S2).

### 2.9. Statistical Analysis

The data were presented as mean ± SEM, based on at least three biological replicates. Statistical comparisons between groups were assessed using either an unpaired Student’s *t*-test or one- or two-way ANOVA using the GraphPad Prism v9.0 platform (Boston, MA, USA). Differences were considered statistically significant at * *p* < 0.05.

## 3. Results

### 3.1. Verapamil Preserves β-Cell Integrity Under Diabetogenic Stress by Modulating Cytotoxicity, Proliferation, and Cell Cycle Progression

Given the established relevance of β-cell dysfunction and loss in the progression of both T1D and T2D, our study focused on examining verapamil’s impact on β-cell viability, proliferation, and cell cycle regulation under high glucose stress. Therefore, to establish the safety profile of verapamil in pancreatic β-cell stress models, verapamil’s cytotoxic profile was assessed under diabetogenic stress by treating MIN6 cells with increasing concentrations of the drug under high-glucose (25.2 mM) culture conditions.

**Figure 1:**
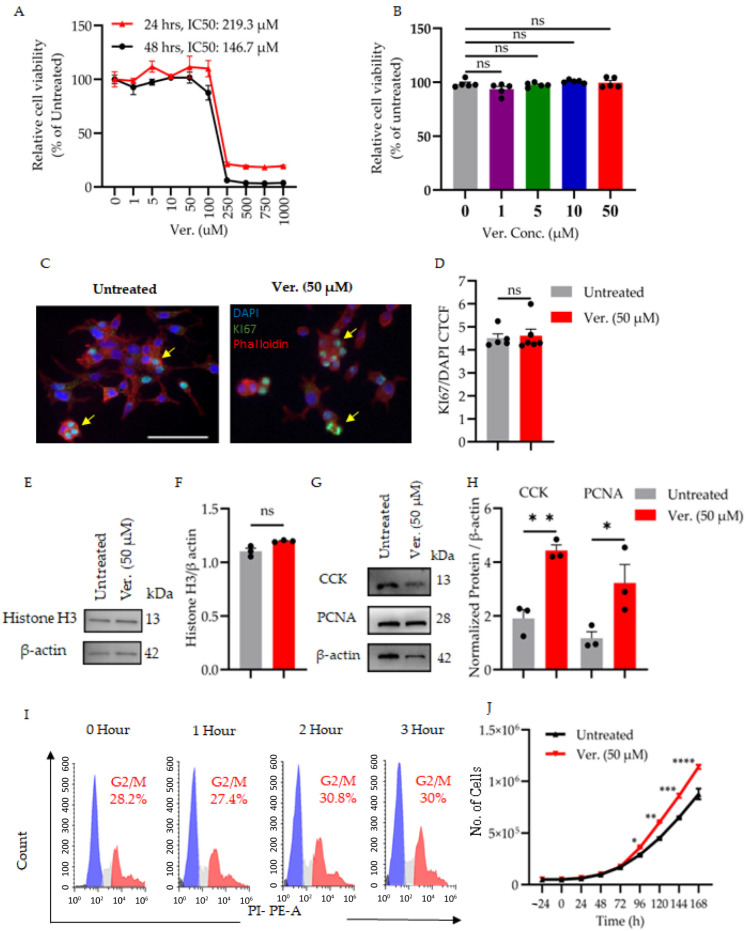
Dose response and proliferative effect of verapamil in MIN6 cells. To determine the proliferative effect of verapamil, (**A**) MIN6 cells were treated with various concentrations of verapamil (μM) for 24 and 48 h in high glucose supplemented DMEM media as described in Section 2. Cell viability percentages were calculated relative to the untreated controls. (**B**) MIN6 cells were cultured in the high glucose supplemented DMEM media in the presence of verapamil (1–50 μM for 24 h), and relative cell viability percentages were calculated for untreated cells. (**C**,**D**) MIN6 cells were cultured on coverslips in high-glucose DMEM media supplemented with 2% free fatty acid BSA. The cells were treated with 50 μM of verapamil for 24 h. Then Ki67 immunostaining was conducted as described in Section 2. Cell compartments were stained as nuclei (blue—DAPI), actin filaments (red—phalloidin), and Ki67 positive proliferating cells (green). Nuclear co-localization is shown by yellow arrows (bar: 50 μm, n = 2). The corrected total cell fluorescence (CTCF) was calculated from 10 fields per sample. (**E**,**F**) Western blotting experiment using proteins extracted from MIN6 cells cultured in high-glucose DMEM media with 2% free fatty acid BSA, treated with 50 μM of verapamil for 24 h to detect the level of histone H3 protein expression as another proliferation marker compared to untreated cells. The level of histone H3 protein was normalized to β-Aactin (n = 2). These data showed no significant changes in relative cell viability, Ki67, and histone H3 protein expressions. (**G**,**H**) MIN6 cells cultured in high-glucose DMEM media with 2% free fatty acid BSA treated with 50 μM of verapamil for 24 h showed significantly higher CCK and PCNA protein expressions relative to β-Actin detected by Western blotting assay (n = 3). (**I**) MIN6 cells were cultured in DMEM media supplemented with FFA-free 2% BSA for 24 h. Then, the cells were treated with verapamil (50 μM) for 1, 2, 3, or 4 h, or left untreated as control. Harvested single cells were fixed with ice-cold 70% ethanol, and after centrifugation, the cells were treated at room temperature (RT) with RNase (100 µg/mL) for 30 min, followed by incubation with PI (40 µg/mL) for another 30 min. DNA content and cell cycle were analyzed using BD FACSCant Flow Cytometer, as described in detail in Section 2. Flow cytometry analysis tracked verapamil’s time-dependent effect on cell cycle phases: dead cells (black), G0/G1 (blue), S-phase (gray), and G2/M (red) cell populations (n = 4 independent experiments). (**J**) MIN6 cells were cultured at a concentration of 5 × 10^4^ cells/well in high-glucose media. The growth curve was plotted by cell counting every 24 h for 7 days, using trypan blue due exclusion assay on the cells treated with verapamil (50 μM, red line) versus untreated cells (black line). Data are presented as mean ± SEM using one-way ANOVA with Tukey’s multiple comparisons test. ns: non-significant, * *p* < 0.05, ** *p* < 0.01, *** *p* < 0.001, **** *p* < 0.0001 versus control.

A dose–response analysis conducted at two different time points revealed distinct time-dependent cytotoxicity. After 24 h of treatment, the half-maximal inhibitory concentration (IC_50_) of verapamil was 219.3 µM, while at 48 h, IC50 increased to 146.7 µM (Figure 1A). These data suggest that although verapamil demonstrates cytotoxic effects at high concentrations, lower concentrations (less than 50 µM) maintain MIN6 cell survival above 85% compared to untreated cells. The toxicity profile becomes more pronounced with extended exposure. This provides guidance for defining a safe dosing window in further experiments. Then, we studied the effect of verapamil on MIN6 β-cell proliferation maintained in supplemented DMEM media containing 25.2 mM glucose for 24 h using an MTT assay. As shown in Figure 1B, cell viability was sustained at these concentrations and was comparable to untreated cells. At the molecular level, the expression of Ki67 (Figure 1C,D and Supplementary Figure S3) and histone 3 (Figure 1E,F), both well-established nuclear markers of active cell proliferation [39], were comparable in both MIN6 cells cultured at 25.2 mM glucose/serum starvation conditions with or without verapamil treatment. Interestingly, the expression levels of CCK and PCNA, markers of cell proliferation, significantly increased in response to verapamil treatment **(**Figure 1G,H). This apparent redundancy in the proliferation markers prompted us to study the cell cycle profile during 3 h of verapamil treatment. We observed a slight time-dependent increase in the proportion of cells undergoing mitosis, as shown by the G2/M phase expansion by cytometric analysis (Figure 1I), indicating a prospective effect of verapamil on MIN6 β-cell proliferation. We next extended the drug treatment for a 7-day time period and monitored cell growth. As shown in Figure 1J, the cell counts of MIN6 cells maintained in supplemented DMEM media containing 25.2 mM glucose showed a significant increase at 96 h of verapamil treatment, relative to untreated cell culture; this increase was further pronounced on days 4–7. Together, these findings indicate that verapamil promotes and stabilizes mitotic activity during periods of metabolic insult and sustains MIN6 β-cell proliferation under hyperglycemia stress conditions.

**Figure 2:**
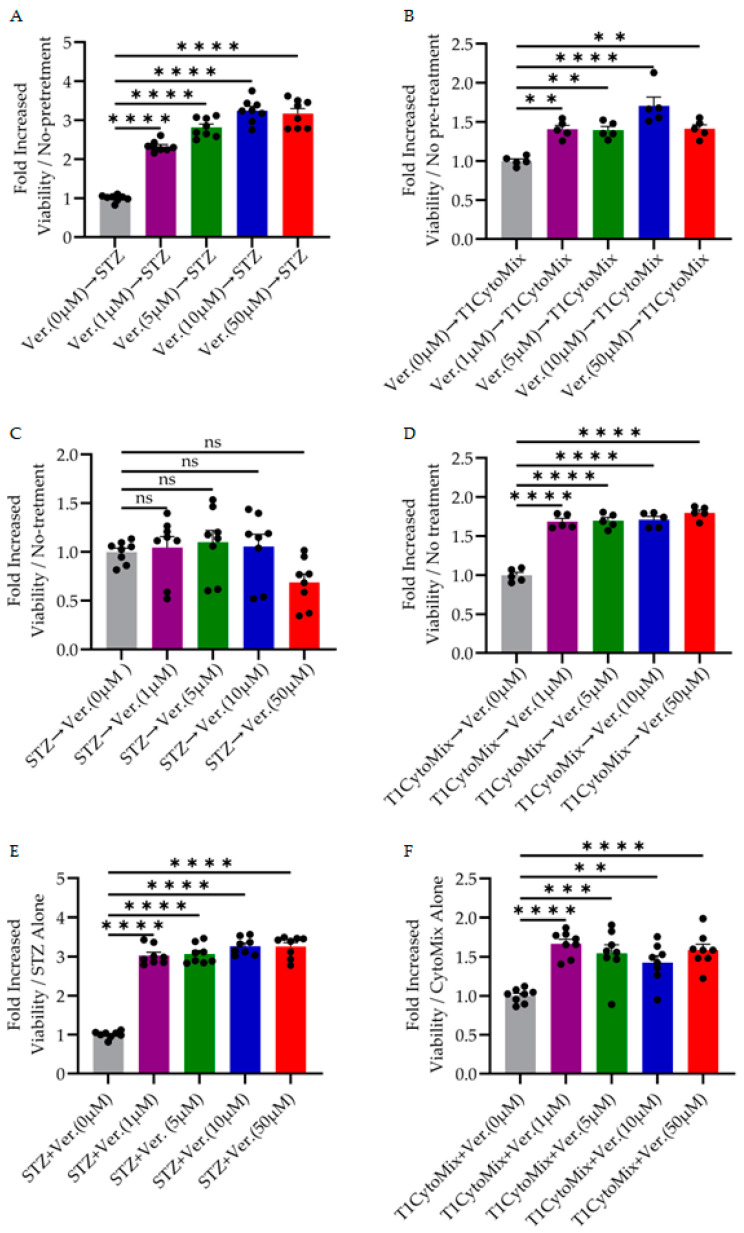
Verapamil protects MIN6 cells against diabetogenic stressors STZ and T1D-cytomix. To detect the protective role of verapamil in MIN6 cells challenged with diabetogenic stressors, MTT assay was conducted on the cells cultured in a high-glucose medium in the presence or absence of stressors (STZ or T1D-cytokine cocktail with different timing of different doses of verapamil (1, 5, 10, and 50 μM) for 24 h under pre-, post-, or co-treatment conditions. (**A**,**B**) Verapamil pre-treatment condition for 24 h, then challenging with STZ or TD-cytomix stressors, respectively. Pre-treatment with verapamil significantly improved cell survival dose-dependently. (**C**,**D**) Verapamil post-treatment enhanced cell recovery moderately after challenge with STZ (**C**) but significantly improved cell survival post-T1D-cytomix cocktail (**D**) treatments for 24 h. (**E**,**F**) Verapamil and stressor co-treatment for 24 h showed significant protection against STZ (**E**) and T1D-cytomix (**F**). Data is presented as the ratio of cell viability of verapamil-treated cells to verapamil-untreated cells. All experiments included >5 biological replicates. Data are presented as mean ± SEM and were analyzed by two-way analysis of variance (ANOVA), and *p* < 0.05 was considered a significant difference. ** *p* < 0.01, *** *p* < 0.001, and **** *p* < 0.0001. ns: non-significant.

Next, we investigated whether verapamil exerts cytoprotective effects in in vitro models of diabetogenic stressors. MIN6 β-cells were exposed to STZ or a T1D-mimicking cytokine mixture (T1D-cytomix: IL-1β, TNF-α, and IFN-γ), with verapamil administered either as pre-treatment, post-treatment, or co-treatment. Our data demonstrates that 24 h verapamil pre-treatment significantly increased MIN6 cell viability in response to both STZ and T1D-cytomix conditions (Figure 2A,B). Notably, verapamil pre-treatment of STZ-challenged cells showed a dose-dependent increase at all concentrations (1–50 μM), producing statistically significant, 2.5- to 3.5-fold, protection compared to STZ-only controls (*p* < 0.0001, Figure 2A). Similarly, in T1D-cytomix-challenged cells, verapamil pre-treatment yielded significant cyto-protection, with the most prominent effects at 10 μM (*p* < 0.0001 for 10 μM, Figure 2B).

Post-treatment with verapamil was largely ineffective after STZ application (Figure 2C), which could be attributed to cell death. On the other hand, verapamil post-treatment of T1D-cytomix-treated cells (Figure 2D) showed a 1.5-fold increase in cellular viability and proliferation after the cytotoxic insult. The indication was comparable at all concentrations used in the study. Furthermore, our data confirms that co-treatment with verapamil during the period of STZ or T1D-cytomix exposure was effective in preserving β-cell viability (Figure 2E,F). With STZ treatment (Figure 2E), all doses of verapamil provided protection (3-fold), with significant increases in cell survival compared to STZ-only controls (*p* < 0.0001). In the case of T1D-cytomix-challenged cells, verapamil co-treatment produced a significant increase in cellular viability, with comparable protection observed at 1 to 50 μM concentrations (*p* < 0.0001, Figure 2F).

Collectively, this dataset supports a strong dose- and kinetically dependent cytoprotective role of verapamil in β-cells exposed to either chemical (STZ) or inflammatory (T1D cyto-mix) stressors under high-glucose conditions. While pre-treatment and co-treatment were consistently effective, post-treatment alone did not restore viability, particularly in STZ model conditions, suggesting that verapamil not only prepares cells to resist injury but also sustains viability during ongoing stress.

### 3.2. Verapamil Enhances Mitochondrial Respiration and Preserves Bioenergetic Function in β-Cells Under Diabetogenic Stress

**Figure 3:**
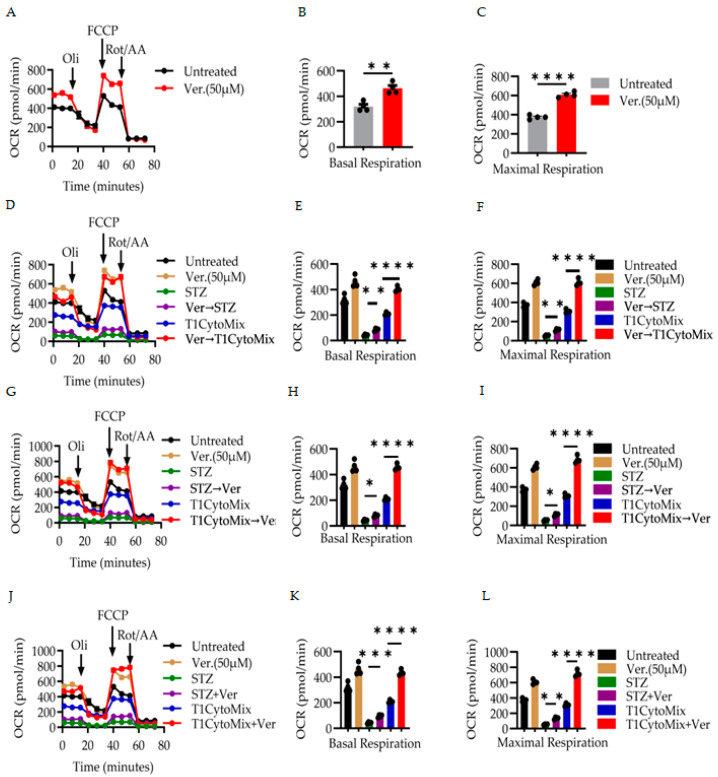
Verapamil enhances mitochondrial function in stressed MIN6 cells. To assess mitochondrial function, MIN6 cells were grown in a high-glucose medium and exposed to either diabetogenic stressors (STZ or a T1D-cytokine mix) with different timings of 50 µM verapamil treatment under pre-, post-, or co-treatment conditions. The cellular oxygen consumption rate (OCR) was measured by metabolic flux analysis following sequential treatment with 1 µM oligomycin, 2 µM FCCP, and 0.5 µM rotenone/antimycin A. (**A**–**C**) Verapamil alone: 50 μM verapamil (24 h) increased both basal (**B**) and maximal (**C**) OCR compared to the untreated controls. (**D**–**F**) Pre-treatment: cells pretreated with verapamil before stress exposure maintained higher basal respiration (**E**) and maximal respiration (**F**). (**G**–**I**) Post-treatment: verapamil administration after stress improved basal OCR (**H**) and maximal OCR (**I**). (**J**–**L**) Co-treatment: simultaneous verapamil + stressor treatment preserved basal respiration (**K**) and maximal respiration (**L**). The OCR data were normalized to the total protein extract measurements. The experimental groups were compared using two-way analysis of variance (ANOVA), with a statistically significant set at *p*-values < 0.05 considered statistically significant, and n = 4 per group. * *p* < 0.05, ** *p* < 0.01, *** *p* < 0.001, and **** *p* < 0.0001.

To investigate the influence of verapamil on mitochondrial respiration, we measured the OCR using metabolic flux analysis. Our data demonstrates that exposure to verapamil (50 μM) resulted in a significant elevation in both basal and maximal respiration relative to untreated controls (Figure 3A–C). Quantitative analysis confirmed a robust increase in basal OCR (*p* < 0.01, Figure 3B) and a more pronounced increase in FCCP-stimulated maximal respiration (*p* < 0.0001, Figure 3C), indicating augmented mitochondrial capacity.

Next, in the context of diabetogenic stress, we explored whether verapamil pre-treatment confers mitochondrial protection (Figure 3D–F). Cells exposed to STZ or the pro-inflammatory T1D-cytomix exhibited marked suppression of OCR (Figure 3D). Remarkably, verapamil pre-treatment (Ver → STZ and Ver → T1D-cytomix) significantly restored both basal and maximal respiratory parameters (*p* < 0.01 for STZ, and *p* < 0.0001 for T1D-cytomic, Figure 3E,F), suggesting a cytoprotective role for verapamil if administered prophylactically [40].

Importantly, when verapamil was administered post-stress, it exerted therapeutic efficacy. In both STZ → Ver and T1D-cytomix → Ver experiments (Figure 3G–I), verapamil post-treatment significantly enhanced mitochondrial respiration compared to stress-only conditions, with statistical significance observed for both basal and maximal respirations (*p* < 0.05 for STZ and *p* < 0.0001 for T1D-cytomix, Figure 3H,I).

In a final set of conditions (Figure 3J–L), co-administration of verapamil with cytotoxic insults (STZ + Ver and T1D-cytomix + Ver) also significantly improved mitochondrial respiratory function, further reinforcing its potential to mitigate stress-induced bioenergetic dysfunction (*p* < 0.001 for STZ and *p* < 0.0001 for T1D-cytomix, Figure 3J,L).

Collectively, these results demonstrate that verapamil enhances mitochondrial oxidative metabolism and preserves β-cell bioenergetics under both preventive and therapeutic frameworks in models of diabetes-relevant cellular stress.

### 3.3. Verapamil Protects β-Cell in Zebrafish Model Against MTZ Stressor

We utilized an in vivo zebrafish model to recapitulate microenvironmental cues that could not be represented in the above MIN6 cell culture model. Zebrafish spontaneously regenerate damaged organs and tissues. Here, we used transgenic Ins:NfsB-mCherry reporter zebrafish larvae to study whether verapamil could replenish or abolish the cytotoxic effect of MTZ on pancreatic β-cells pool.

**Figure 4:**
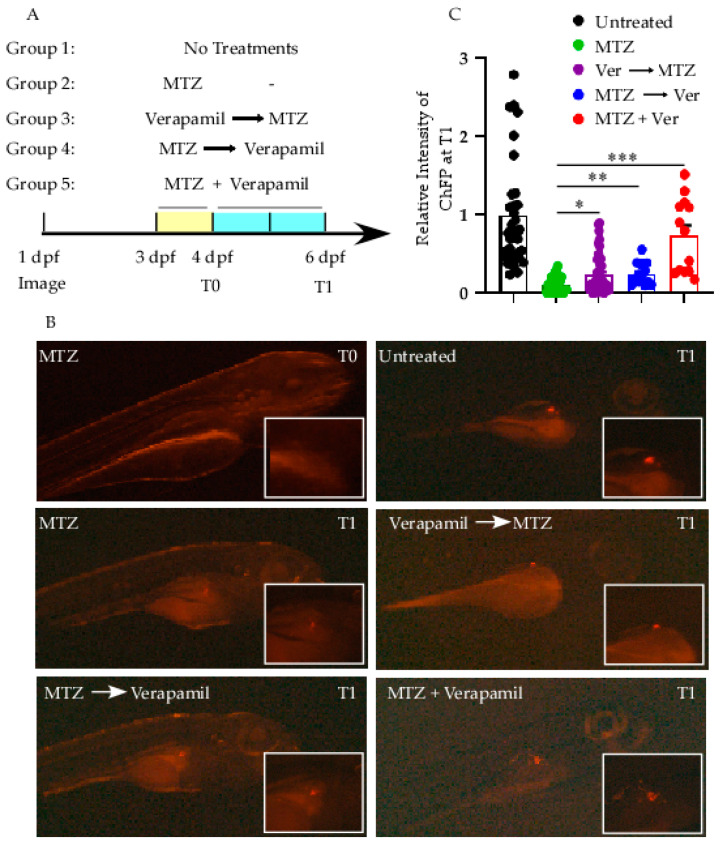
Verapamil treatments overcome the cytotoxic effect of MTZ in zebrafish larvae. (**A**) Experimental timeline and treatment groups. Larvae (3 dpf) were randomly divided into five groups: Group 1 (untreated), normal developmental control; Group 2 (MTZ alone), larvae exposed to 10 mM MTZ for 24 h (to damage β-cells); Group 3 (Verapamil → MTZ), pre-treatment with 10 µM verapamil for 24 h before 10 mM MTZ exposure for 48 h; Group 4 (MTZ → Verapamil), post-treatment with 10 µM verapamil for 48 h after 10 mM MTZ insult for 24 h; Group 5 (MTZ + Verapamil), co-treatment with both 10 µM verapamil and 10 mM MTZ for 72 h. mCherry fluorescence reporter protein (ChFP) expressed in the larvae’s β-cells was imaged at 4 dpf (T0) and 6 dpf (T1). The change in ChFP intensity between T0 and T1 was measured. (**B**) Representative images of β-cells (red fluorescence) at T0 post-MTZ damage and T1 at recovery phase. Inserts show the magnified ChFP area. (**C**) Quantification of β-cell fluorescence (ChFP) at T1. Untreated Group 1 showed the physiological development of β-cells. The MTZ-alone group, Group 2, showed severe loss of fluorescence at T1, whereas the verapamil-treated groups 3–5 showed an improved β-cell preservation or recovery. Images were taken using Stereo Discovery 1.2 ZIESS microcopy. Experiments were performed in three independent trials (n = 20–30 larvae/group). Data are presented as mean ± SEM values. * *p* = 0.016, ** *p* = 0.01, *** *p* = 0.0004.

In comparison to larvae treated with MTZ alone (Group 2), pre-treatment with verapamil (Group 3) showed a significantly higher fluorescence intensity (*p* = 0.016, Figure 4B,C). Similarly, post-treatment with verapamil (Group 4) also alleviated the fluorescence intensity significantly (*p* = 0.01, Figure 4B,C). Notably, co-treatment with verapamil and MTZ mitigated the cytotoxic effect of MTZ (Group 5; *p* = 0.0004, Figure 4B,C). These observations indicate not only that verapamil promotes cell viability and proliferation in the presence of the MTZ cytotoxic effect, but also that it neutralizes and reduces MTZ’s adverse effects on pancreatic β-cells in zebrafish larvae.

## 4. Discussion

Our findings highlight the substantial multifaced potential of verapamil to protect pancreatic β-cells under diabetogenic stress, such as cellular stress, mitochondrial dysfunction, and cell death. By administrating diabetogenic stress and metabolic analyses in cell and zebrafish models, we demonstrated that verapamil could be a valuable preventive or therapeutic strategy in diabetes.

The deterioration of functional β-cell mass is a central event in the onset and progression of diabetes. This process is primarily mediated by prolonged exposure to elevated levels of blood sugar, oxidative stress, and proinflammatory cytokines [41,42,43]. Unfortunately, few existing drugs directly protect or restore β-cell mass, making new approaches urgently needed. Verapamil, a widely used L-type calcium channel blocker as a blood pressure medication, has emerged as a promising candidate [31,44].

Mechanistically, verapamil treatment elevated the expression of CCK, a well-characterized incretin hormone typically induced by GLP-1 signaling [45]. Here, we confirm our previous observation of CCK induction following verapamil, even under high-glucose conditions and in the presence of various cytokines [20]. CCK exhibits protective roles in the endocrine pancreas, particularly in preserving β-cell mass with age and protecting them from apoptosis triggered by diabetogenic insults such as STZ and pro-inflammatory cytokines [46]. In our experiments, the cytoprotective effects of verapamil against STZ and T1D-cytomix may be, at least in part, mediated through CCK upregulation. Furthermore, our previous study indicated that verapamil reshapes the transcriptomic and proteomic landscape of MIN6 cells, engaging multiple pathways that converge on stress mitigation, mitochondrial preservation, and redox balance [20]. A key molecular event is the downregulation of the thioredoxin-interacting protein (TXNIP), a redox-sensitive protein linked to β-cell apoptosis and oxidative stress [47]. Reduced TXNIP expression alleviates thioredoxin inhibition, decreases reactive oxygen species, and suppresses inflammasome activation, thereby preventing β-cell death. Furthermore, verapamil increases Wnt4 expression, a calcium signaling modulatory ligand involved in mitochondrial activity and redox balance. Activated Wnt4 enhances MIN6 cell growth and insulin vesicle transport, resulting in improved secretory capacity [23]. The drug also activates CaMK4, a calcium/calmodulin-dependent kinase that triggers the CREB–IRS2 pathway [22], promoting proliferation and anti-apoptotic characteristics in the MIN6 cells. These actions are supplemented by verapamil’s suppression of VDAC1/2 expression, mitochondrial permeability, and apoptosis-related proteins [48], thereby stabilizing mitochondrial function and enhancing ATP-linked insulin secretion. Collectively, these molecular alterations amount to increase β-cell resistance to glucotoxic, lipotoxic, and pro-inflammatory stress. By modulating calcium-dependent signaling, redox protection, and mitochondrial homeostasis, verapamil promotes β-cell survival, regeneration, and functional integrity. These findings provide mechanistic justification for the fact that the broadly accepted calcium channel blocker verapamil is a promising repurposed therapeutic candidate for preserving β-cell mass and slowing progression to diabetes.

In addition, verapamil’s benefits may go beyond just preventing cell apoptosis. Calcium signaling, which verapamil influences, plays a crucial role in β-cell physiology, including ER stress, mitochondrial function, and insulin exocytosis [49]. By modulating calcium flow, verapamil may reduce harmful signaling cascades that cause deterioration in diabetes. This mechanism aligns with the observed improvements in mitochondrial function in stressed cells and provides further support for calcium signaling as a therapeutic axis [50].

Animal research also hints at verapamil’s ability to promote β-cell regeneration. In zebrafish, the pancreas exhibits a regenerative response following insult, making it an ideal system for evaluating pro-regenerative compounds [51]. Our in vivo data showed improved β-cell mass recovery, suggesting that verapamil may activate endogenous repair mechanisms, possibly by boosting β-cell proliferation and dedifferentiation/re-differentiation cycles, or attenuating inflammation [52]. Although zebrafish regeneration differs from mammalian repair, these findings suggest that verapamil might support repair mechanisms, particularly when endogenous regenerative programs are still active in early diabetes [40,53,54].

Another key discovery is verapamil’s positive impact on mitochondrial oxidative phosphorylation, which highlights a new dimension of its therapeutic potential. Since β-cells depend on mitochondrial metabolism to couple glucose sensing with insulin release, restoring their metabolic function could be crucial in preventing diabetes progression [55,56]. Verapamil’s ability to enhance mitochondrial activity opens up new therapeutic possibilities worth exploring further.

Our data suggests that earlier verapamil administration works best, either before or alongside diabetogenic insults. Post-injury treatment exhibited variable efficacy, particularly in the STZ model. This divergence may reflect the irreversible cellular damage incurred prior to drug exposure, underlining the importance of early intervention. In contrast, the partial efficacy observed in the T1D-cytomix model implies that verapamil may retain therapeutic value even post-immune activation, potentially through modulating the apoptotic or metabolic pathways. Notably, our findings are already translating into real-world benefits. A clinical trial by Ovalle et al. reported that oral verapamil administration in patients with recent-onset T1D maintained their insulin secretion for at least a year [13]. Follow-up studies confirmed that verapamil reduced TXNIP and inflammatory markers [57,58], mirroring earlier results in mice [59,60,61]. Together, these studies suggest that verapamil could be re-purposed to help preserve β-cells in diabetes.

## 5. Conclusions

Overall, verapamil’s safety, affordability, and multiple protective mechanisms, such as calcium regulation, mitochondrial support, anti-inflammatory effects, and cell resilience, make it a strong candidate for diabetes therapy. It could be especially valuable in low-resource settings or as an add-on to current treatments.

Once-daily oral verapamil, in combination with standard insulin therapy, has already demonstrated an acceptable safety profile, with no major adverse events reported. The incorporation of immunomodulatory approaches into future trials can further maximize its therapeutic value.

Clarification of the molecular targets for verapamil may also reveal more universal pathways that are susceptible to targeting β-cell salvage or to designing second-generation therapeutic analogues. T1D interventional therapy trials for restoring functional β-cell mass are critically needed.

International collaboration between diabetes research groups and drug regulatory authorities will be necessary to develop consistent guidelines and best practices for the safe and effective provision of verapamil therapy in diabetes management.

While verapamil treatments are promising, the exact mechanisms that help β-cells regenerate and maintain their energy production remain elusive. Future studies should investigate the molecular mechanisms behind these protective effects, including signaling pathways responding to verapamil administration, potential off-target effects, modulation of ER stress signaling pathways, antioxidant defense systems, and regulation of pro-survival transcriptional programs.

Most importantly, large and long-term clinical trials in patients with diabetes, including diverse populations, are needed to confirm the protective role of verapamil over time and its broader efficacy. Ancillary animal model studies should incorporate β-cell quantitation, proliferation, and apoptosis assays, as well as glucose tolerance analyses, to evaluate both mechanistic and functional endpoints in vivo.

A key limitation of this study is the use of zebrafish embryos, which restricts the ability to directly measure insulin secretion and perform detailed glucose homeostasis studies. Adult zebrafish may provide a more physiologically relevant model for evaluating glucose metabolism, β-cell function, and the long-term effects of verapamil. Additionally, zebrafish embryos have immature pancreatic architecture and limited β-cell mass, which may not fully recapitulate human diabetes physiology. Other constraints include differences in drug absorption, metabolism, and off-target effects between zebrafish and mammals, which may limit the direct translatability of the findings to human patients.

While previous studies have been predominantly performed in patients with recent T1D onset, it will be important to determine if verapamil is also effective in patients with established diabetes, prediabetes, or metabolic syndrome—cohorts at high risk of β-cell failure.

## Data Availability

The original contributions presented in this study are included in the article/Supplementary Material. Further inquiries can be directed to the corresponding authors.

## Supplementary Materials

The following supporting information can be downloaded at https://www.mdpi.com/article/10.3390/cells14211695/s1.
