# Supplementary material for: Verapamil Restores β-Cell Mass and Function in Diabetogenic Stress Models via Proliferation and Mitochondrial Respiration
Source: Cells. 2025 Oct 29;14(21):1695. doi: 10.3390/cells14211695 (PMC12610477; doi:10.3390/cells14211695)

Supplementary Figure S1

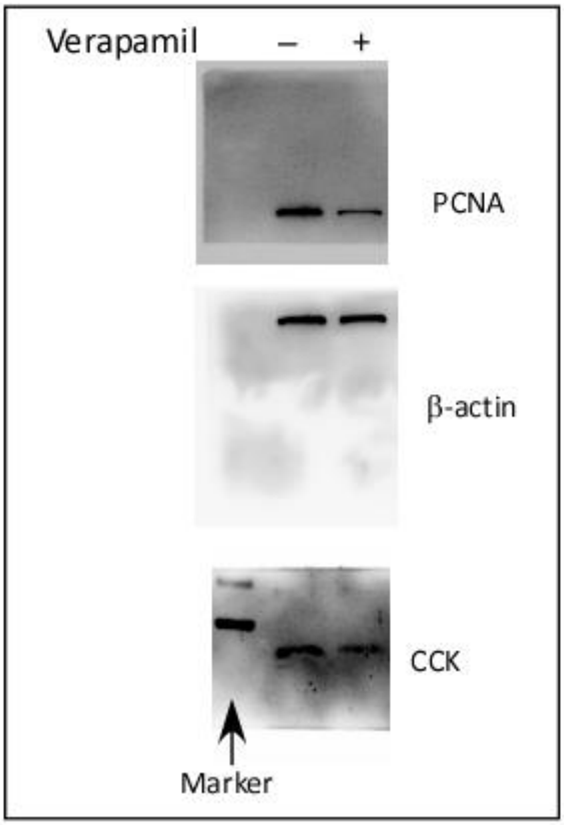

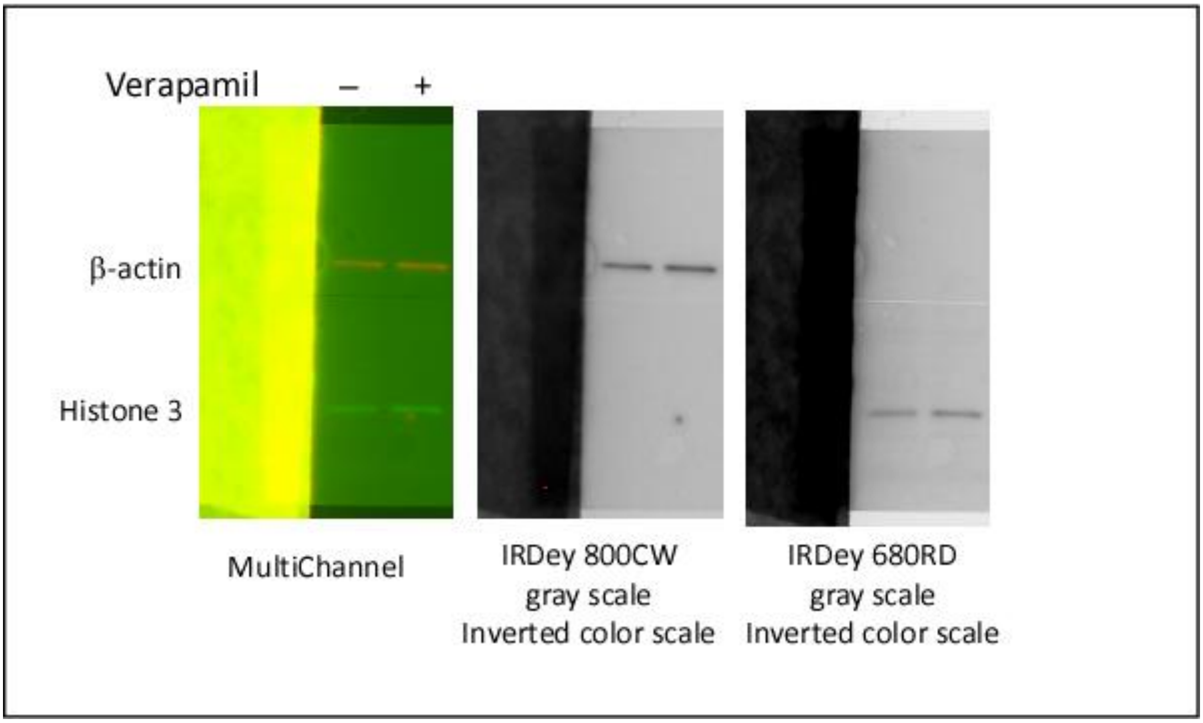

Supplementary Figure S2

ImageJ Analysis Algorithm

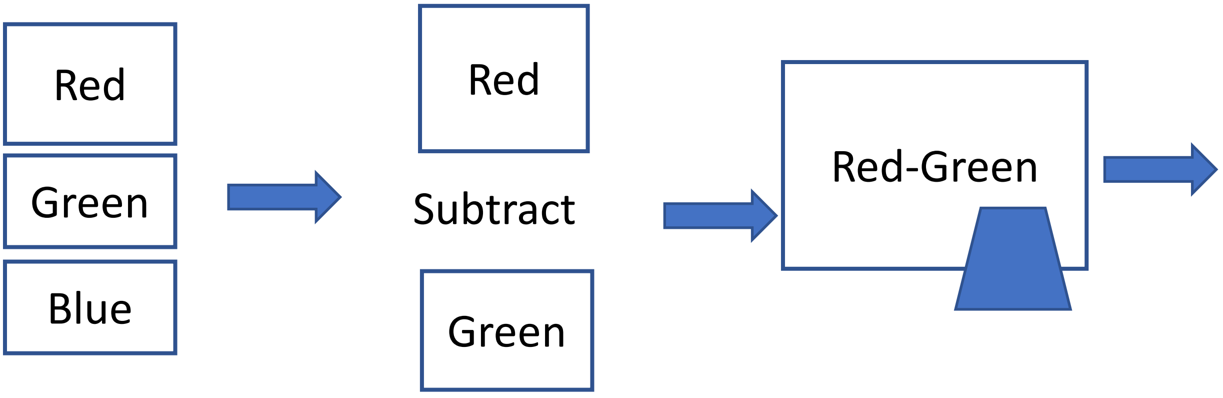

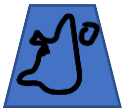

```
run("Duplicate...", " ");
waitForUser("select area click OK");
run("Clear Outside");
setAutoThreshold("Default dark");
//run("Threshold...");
setThreshold(455, 65535, "raw");
run("Create Selection");
run("Put Behind [tab]");
run("Restore Selection");
run("Measure");
waitForUser("click OK if you have more");
run("Open Next");
run("Put Behind [tab]");
close();
run("Select None");

```

This script separates Red/Green/Blue and removes the Blue. It subtracts Green, which is the background, from the Red. Then, the correct region is selected with rough selection tool. The software is used to threshold the intensity over a select minimum. A fine selection area is created to generate data for mean intensity and area.

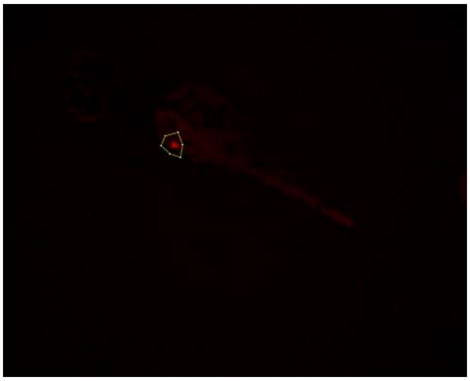

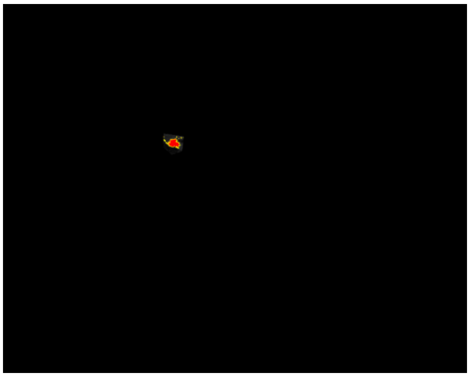

Supplementary Figure S3

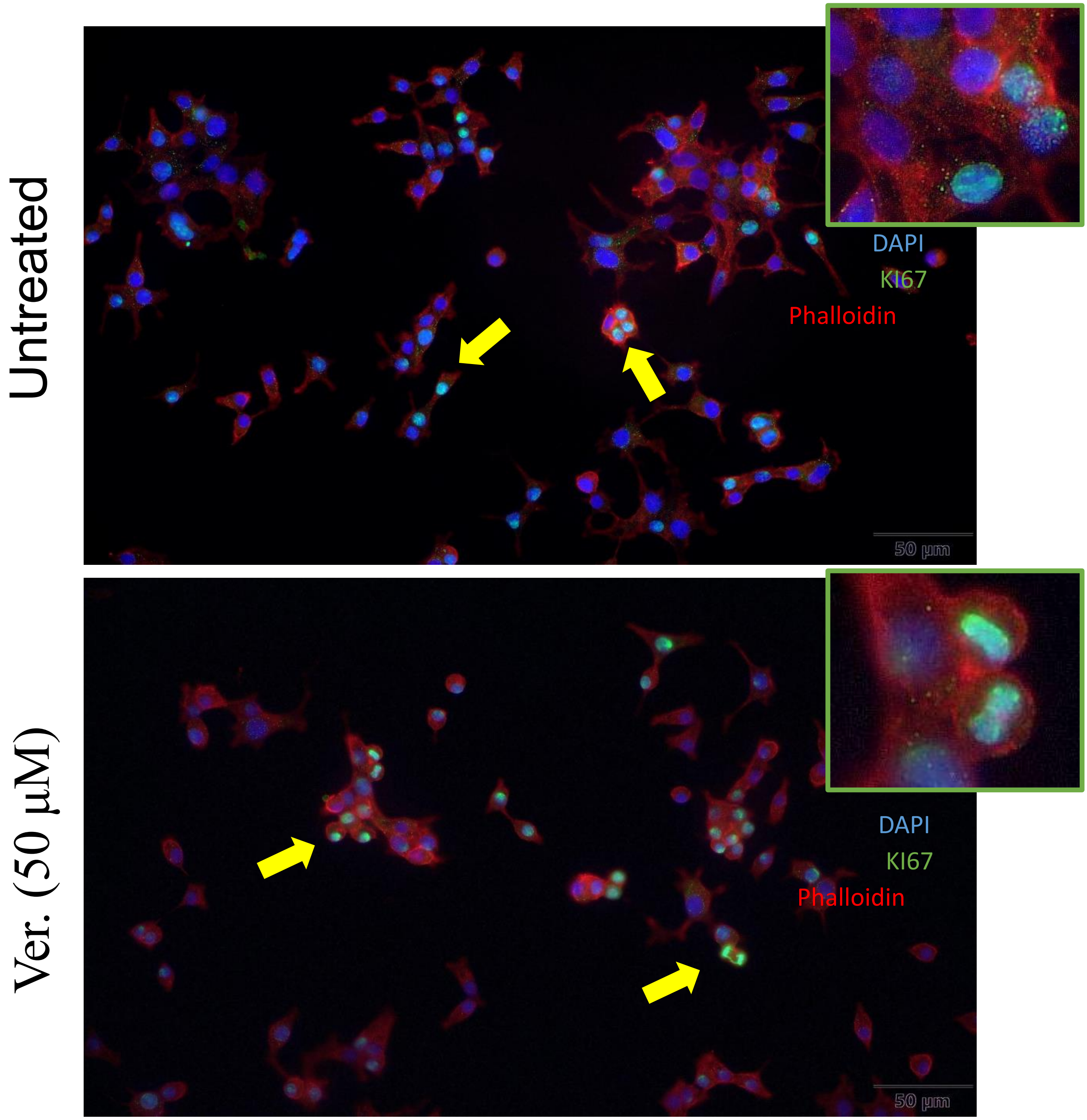

Supplement: Supplementary file 1 [file cells-14-01695-s001.zip › cells-3815433-supplementary.pdf]
